# Small-diameter melanomas (micromelanomas): clinical, dermoscopic and histopathological findings^☆^

**DOI:** 10.1016/j.abd.2020.10.014

**Published:** 2021-11-20

**Authors:** Pablo Vargas-Mora, Rubén González-Cuevas, Leonardo Peruilh-Bagolini, Fernando Valenzuela

**Affiliations:** Department of Dermatology, Faculty of Medicine, Universidad de Chile, Santiago, Chile

Dear Editor,

Melanoma is one of the cancers with the highest rates of increase in the last decades. Traditionally, mass education strategies have emphasized the importance of melanomas of > 6 mm (ABCDE rule).^1^, ^2^ However, the optimization of diagnostic methods, principally using dermoscopy, allows the early identification of melanomas smaller than this (micromelanomas). These may represent up to a third of all melanomas.^3^, ^4^, ^5^ For this reason, we decided to characterize the clinic, dermoscopy, and histopathology of micromelanomas in our institution.

A retrospective study in the Dermatology Department of the Hospital Clínico de la *Universidad de Chile*, with all patients that had a diagnosis of cutaneous melanoma with a clinical diameter up to 5 mm, between January 1^st^, 2003, and December 31, 2018, were included. The following characteristics were evaluated: sex, age, location, clinical diameter, dermoscopy (evaluated by two dermoscopists separately) and clinical diagnosis, as well as histopathological characteristics such as diagnosis (in situ or invasive), Breslow Index (BI), and ulceration. Descriptive statistics were applied using absolute numbers, percentages, averages, and standard deviation.

A total of 20 patients were evaluated (Table 1), 15 women (75%), and the mean age was 50.4 years (±13.4; range 28–79 years). The most common location was the lower limbs (9/20), followed by the head/neck (5/20), trunk (4/20), and upper limbs (2/20). The mean clinical diameter was 3.7 mm (±1.0; range 2–5 mm). The most frequent clinical diagnosis was atypical nevus (9/20), followed by melanocytic nevus (5/20), melanoma (5/20), and seborrheic keratosis (1/20). There was a photographic record of dermoscopy as much as twelve lesions, and an atypical network pattern was found in 6/12 (Fig. 1A and B), followed by irregular dots/globules (3/12), irregular hyperpigmented areas (3/12), and atypical blotches (2/12) (Fig. 2A and B). From the histopathological study: 12 cases (60%) showed in situ melanomas and the other 8 (40%) invasive melanomas, with a BI between 0.25 and 2.8 mm. None of the lesions showed ulceration. In two cases sentinel node biopsy was carried out, with negative results.

**Table 1:** Clinical, dermatoscopic and histopathological features of micromelanomas (n = 20).

Case	Sex/age	Clinical diagnosis	Location	Size (mm)	Histopathological diagnosis	Breslow index (mm)	Dermoscopy: main pattern	Dermoscopy: melanoma criteria
1	F/44	Atypical nevus	Trunk	4	In situ	N/A	Reticular	AN, IHA
2	F/45	Atypical nevus	Upper limbs	4	In situ	N/A	Reticular	AN, IG
3	M/72	Atypical nevus	Trunk	4	Invasive	0.75	Multicomponent	TSA, AB, SWS
4	F/45	Melanoma	Lower limbs	2	In situ	N/A	N/R	N/R
5	M/44	Melanoma	Lower limbs	3	Invasive	1.2	N/R	N/R
6	F/50	Nevus	Lower limbs	5	Invasive	0.25	Reticular	AN
7	F/60	Melanoma	Head/neck	3	In situ	N/A	Reticular	AN
8	M/52	Intradermal nevus	Head/neck	5	Invasive	2.8	N/R	N/R
9	F/61	Nevus	Head/neck	3	In situ	N/A	N/R	N/R
10	M/50	Melanoma	Head/neck	5	Invasive	0.9	Reticular	AN
11	F/79	Nevus	Head/neck	2	Invasive	0.8	N/R	N/R
12	F/30	Atypical nevus	Lower limbs	3	In situ	N/A	N/R	N/R
13	F/40	Atypical nevus	Upper limbs	3	In situ	N/A	Globular	IG
14	F/28	Atypical nevus	Lower limbs	5	In situ	N/A	Bicomponent^a^	IHA
15	F/36	Seborrheic keratosis	Trunk	5	Invasive	0.7	N/R	N/R
16	F/68	Atypical nevus	Lower limbs	3	Invasive	0.4	Structureless	AB
17	F/44	Atypical nevus	Lower limbs	3	In situ	N/A	Reticular	AN
18	F/46	Atypical nevus	Lower limbs	3	In situ	N/A	N/R	N/R
19	M/64	Nevus	Trunk	5	In situ	N/A	Bicomponent^a^	IG
20	F/50	Melanoma	Lower limbs	4	In situ	N/A	Bicomponent^a^	IHA

F, Female; M, Male; N/A, Not Applicable; N/R, Not Registered; AN, Atypical Network; TSA, Tan Structureless Areas; AB, Atypical Blotches; IG, Irregular dots/Globules; SWS, Shiny White Structures; IHA, Irregular Hyperpigmented Areas.

a Bicomponent with the reticular pattern associated.

**Figure 1:**
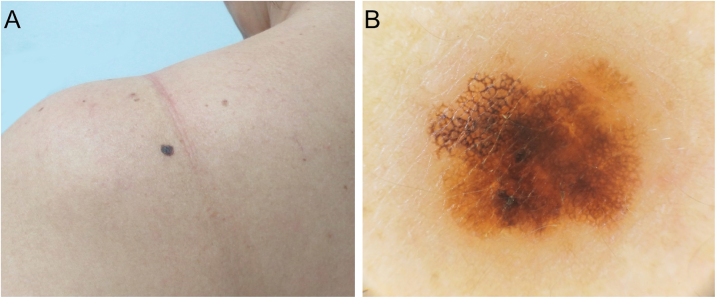
(A), In situ melanoma. Clinical diameter of 4 mm. (B), Dermoscopy shows an atypical network with irregular hyperpigmented areas.

**Figure 2:**
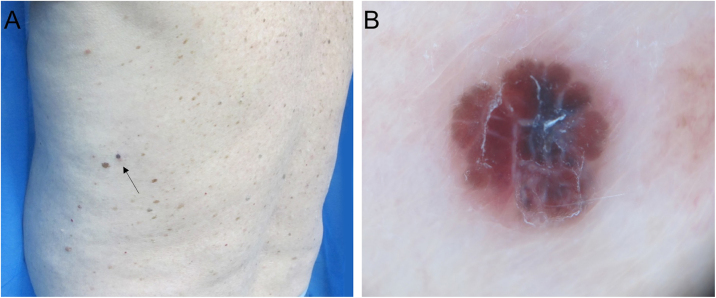
(A), Invasive melanoma of 0.75 mm in Breslow thickness with a diameter of 3 mm. (B), Dermoscopy shows a multi-component pattern with tan structureless areas, atypical blotch, shiny white structures, and serpentine vessels.

The present study is the first series of micromelanomas in Latin American patients. We point out the high percentage of invasive melanomas, including one case with a BI over 2 mm. This is similar to that shown in other studies which have reported between 27% and 45% invasive melanomas.^1^, ^3^, ^6^ However, Bono et al., reported an invasive component in 19 of their 23 lesions of 3 mm or less (83%). This is highly noteworthy and worrying, given that it contrasts with classical thinking which suggests that small lesions are early-stage lesions.^2^

Strategies for the early detection of melanomas, like the ABCDE rule, are not very efficient for recognizing micromelanomas. A clinical characteristic suggested by several authors is the greater frequency and intensity of black coloration. This suggests the possibility of changing the ABCDE rule, with ‘Dark’ replacing ‘Diameter’ as the letter ‘D’ for these lesions.^6^ However, it should be noted the poor utility of this rule in nodular melanomas, which may represent an important part of thicker micromelanomas.

As for the dermoscopy, we observed a more frequent atypical network, irregular dots/globules, and irregular hyperpigmented areas, as described in the literature.^3^, ^7^ According to Seidenari et al., micromelanomas lack many of the characteristics that larger lesions have, as they are less asymmetrical, have fewer colors, and have a lower frequency of a regression. They also have practically no atypical vessels or blue-white veil, and this undoubtedly makes diagnosis more difficult.^3^

Small diameter melanocytic lesions should be evaluated in the same way as larger lesions, because if they turn out to be melanomas the possibility, they will be invasive is not low, as our study shows.

## Financial support

None declared.

## Authors’ contributions

Pablo Vargas-Mora: Approval of the final version of the manuscript; conception and planning of the study; elaboration and writing of the manuscript; obtaining, analyzing, and interpreting the data; effective participation in research orientation; critical review of the literature; critical review of the manuscript.

Rubén González-Cuevas: Approval of the final version of the manuscript; conception and planning of the study; elaboration and writing of the manuscript; obtaining, analyzing, and interpreting the data; effective participation in research orientation; critical review of the literature; critical review of the manuscript.

Leonardo Peruilh-Bagolini: Approval of the final version of the manuscript; conception and planning of the study; elaboration and writing of the manuscript; obtaining, analyzing, and interpreting the data; effective participation in research orientation; critical review of the manuscript.

Fernando Valenzuela: Approval of the final version of the manuscript; conception and planning of the study; obtaining, analyzing, and interpreting the data; effective participation in research orientation; critical review of the manuscript.

## Conflicts of interest

None declared.
